# Web-based guided self-help cognitive behavioral therapy–enhanced versus treatment as usual for binge-eating disorder: a randomized controlled trial protocol

**DOI:** 10.3389/fpsyt.2024.1332360

**Published:** 2024-02-16

**Authors:** Ella van Beers, Bernou Melisse, Margo de Jonge, Jaap Peen, Elske van den Berg, Edwin de Beurs

**Affiliations:** ^1^ Novarum Center for Eating Disorders & Obesity, Amstelveen, Netherlands; ^2^ Utrecht University, Department of Clinical Psychology, Utrecht, Netherlands; ^3^ Department of Research, Arkin Mental Health Institute, Amsterdam, Netherlands; ^4^ Leiden University, Department of Clinical Psychology, Leiden, Netherlands

**Keywords:** binge eating disorder, guided self-help, cognitive behavioral therapy-enhanced, web-based treatment, randomized controlled trial

## Abstract

Binge-eating disorder (BED) is a psychiatric disorder characterized by recurrent episodes of eating a large amount of food in a discrete period of time while experiencing a loss of control. Cognitive behavioral therapy-enhanced (CBT-E) is a recommended treatment for binge-eating disorder and is typically offered through 20 sessions. Although binge-eating disorder is highly responsive to CBT-E, the cost of treating these patients is high. Therefore, it is crucial to evaluate the efficacy of low-intensity and low-cost treatments for binge-eating disorder that can be offered as a first line of treatment and be widely disseminated. The proposed noninferiority randomized controlled trial aims to determine the efficacy of web-based guided self-help CBT-E compared to treatment-as-usual CBT-E. Guided self-help will be based on a self-help program to stop binge eating, will be shorter in duration and lower intensity, and will require fewer therapist hours. Patients with binge-eating disorder (N = 180) will be randomly assigned to receive guided self-help or treatment-as-usual. Assessments will take place at baseline, mid-treatment, at the end of treatment, and at 20- and 40-weeks post-treatment. Treatment efficacy will be measured by examining the reduction in binge-eating days in the previous 28 days between baseline and the end of treatment between groups, with a noninferiority margin (Δ) of 1 binge-eating day. Secondary outcomes will include full remission, body shape dissatisfaction, therapeutic alliance, clinical impairment, health-related quality of life, attrition, and an economic evaluation to assess cost-effectiveness and cost-utility. The moderators examined will be baseline scores, demographic variables, and body mass index. It is expected that guided self-help is noninferior in efficacy compared to treatment-as-usual. The proposed study will be the first to directly compare the efficacy and economically evaluate a low-intensity and low-cost binge-eating disorder treatment compared to treatment-as-usual. If guided self-help is noninferior to treatment-as-usual in efficacy, it can be widely disseminated and used as a first line of treatment for patients with binge-eating disorder. The Dutch trial register number is R21.016. The study has been approved by the Medical Research Ethics Committees United on May 25th, 2021, case number NL76368.100.21.

## 1 Introduction

Binge-eating disorder (BED) is a serious psychiatric disorder with detrimental effects on physical and mental health. Patients with BED engage in recurrent episodes of eating abnormally large amounts of food in discrete periods of time while feeling a lack of control over their actions (1). Patients with BED frequently eat until they are uncomfortably full and experience elevated levels of distress around their behavior (1). The *Diagnostic and Statistical Manual of Mental Disorders – 5^th^ Edition* (2) states that for a diagnosis of BED, binge eating must occur at least one day per week for three months, and the binges must not be associated with compensatory behaviors such as self-induced vomiting, excessive exercise, or fasting. BED is more common than anorexia nervosa and bulimia nervosa combined (3), with a lifetime prevalence of 1.9% in upper- and high-income countries (4, 5), however the actual prevalence of BED is likely much higher given many individuals who meet BED criteria never get a formal diagnosis or receive treatment (6). Approximately 30% of patients with BED have excess weight, and 32% are classified as having obesity (classes 1 – 3), resulting in many serious health conditions (4).

Many BED patients have secondary health complications including type II diabetes, hypertension, dyslipidemia, heart disease, and stroke (4, 7). Psychiatric comorbidities are seen in up to 80% of patients with BED, with many patients having increased rates of anxiety disorders, major depressive disorder, obsessive-compulsive disorder, and bipolar I and II (8). Patients with BED also score poorer on quality of life measures due to excess weight and higher levels of depression and anxiety (9).

The impact of BED on healthcare utilization is significant; many patients with BED receive inappropriate care through weight-loss programs and engage in treatments aimed at addressing their psychological comorbidities (4, 10) rather than their eating disorder as the root problem. The economic cost of BED is substantial; in the United States, the one-year general healthcare cost for those with BED was $18,152 higher than those without an eating disorder (in estimated 2014 USD; 11). Another study from the USA found that people with BED have higher generic healthcare costs by approximately $2,758 per year compared with their age- and gender-matched means (in estimated 2009 USD; 12). In the UK, the yearly cost of health services per eating disorder patient is £8,850, with an additional £19,700 in direct and indirect financial costs to patients and their caregivers (13). Such figures are unknown for Dutch patients and caregivers; however, these figures are likely similar in the Netherlands, highlighting the need for effective, efficient, and low-cost treatments for BED.

Cognitive behavioral therapy-enhanced (CBT-E; 14), is an evidence-based treatment for BED (15–17). CBT-E uses a transdiagnostic approach for the treatment of eating disorders and takes a highly individualized approach (12). The treatment is broken down into four stages to strategically address disturbances in eating behaviors, establish a normalized eating pattern, and promote new ways of coping (12). CBT for eating disorders can be delivered in person, through guided self-help (18–22) or self-help with minimal support and guidance from a therapist (23), video conferencing (24), or a recently developed web-based GSH program (21, 25). Given CBT has several methods of delivery, it is important to evaluate whether a stepped-care model (e.g. providing patients with low-cost and low-intensity treatment first before referring to specialized or more intensive care) is feasible, whereby less-intensive treatments such as web-based GSH are offered as a first-line of treatment (16).

The stepped-care model, or firstly referring patients for low-intensive care and increasing the level of care as needed, is ideal from an economic and healthcare perspective, as it can decrease the need for costly and specialized services, reduce treatment waiting times compared with more conventional treatments (26), and may improve long-term patient outcomes (27, 28). The stepped-care model is also preferable for patients as there is less social disruption, less or no need to take time off from school or work, geographical barriers are removed, and patients can have increased autonomy (29, 30). The stepped-care model is recommended for BED in the United Kingdom and Australia, whereby GSH is recommended as the first line of treatment (15, 16). However, despite the potential cost savings in the Netherlands (21, 31, 32) and long waiting lists for treatment, the Netherlands continues to recommend in-person therapy as a first line of treatment (33). Therefore, it is critical to examine whether a lower-intensity and remotely accessible treatments such as GSH and web-based GSH are viable for patients with BED. If GSH is offered as a first line of treatment, patients will benefit from shorter waitlists, improved health and quality of life, and the healthcare burden from BED can be mitigated (34, 35).

The first version of CBT-E self-help was developed by Christopher Fairburn (18, 36) in the book *Overcoming Binge Eating*, a self-help book comprised of two parts: Part I offers a description of binge eating and why it occurs, and Part II provides a CBT-E-based self-help program to stop binge eating. The book is intended for anyone with a binge-eating problem, as long as they are not significantly underweight (36). Studies have suggested GSH can empower patients to take ownership and accountability over their eating disorder recovery (37, 38), leading to a more robust recovery. Furthermore, a review on self-help and GSH for eating disorders suggests that patients using these modalities learn to manage binge eating without relying on external supports, which may better support them in the long-term to handle setbacks. If patients learn and develop the skills that they need to manage their binge eating on their own without relying on external supports, they may be better equipped to handle setbacks (37). A recent randomized controlled trial comparing web-based GSH CBT-E with a waitlist condition found that the treatment reduced the number of objective binges from an average of 19 (*SD* = 16) at the start-of-treatment to 3 (*SD* = 5) at the end of treatment, with an interaction effect between time and condition (treatment or waitlist; *F2*,178 = 18.55; *P*<.001) (21). These improvements were sustained at 12- and 24-week follow-ups (21). These findings highlight the importance of not only GSH, but remotely accessible GSH treatments for BED, which could be offered to patients who lack access to treatment or do not have a formal diagnosis, While web-based GSH CBT-E offers many advantages, it is unknown whether it is as efficacious as treatment as usual CBT-E. This is critical in determining whether web-based GSH is a viable alternative that can be offered as a first line of treatment for BED.

The present study aims to determine whether web-based GSH CBT-E is noninferior in efficacy compared to treatment as usual CBT-E (TAU CBT-E) for treating BED. Efficacy will be examined by the reduction of binge-eating days, defined as the number of days on which one or more binge-eating episodes occurred, at the end of treatment. This study sought to determine whether web-based GSH CBT-E is noninferior in efficacy compared to TAU CBT-E in reducing the number of binge-eating days at the end of treatment. In addition to examining the reduction in binge-eating days, several secondary outcomes will also be evaluated, including full remission, body shape dissatisfaction, therapeutic alliance, clinical impairment, health-related quality of life, attrition, and an economic evaluation to assess the cost-effectiveness and cost-utility of web-based GSH CBT-E compared to TAU CBT-E (described below). Moderators will be examined, including baseline scores, demographic variables, and body mass index (BMI). It is expected that participants in both groups will have a reduction in the number of binge-eating days, with web-based GSH CBT-E being noninferior to TAU CBT-E.

## 2 Method

### 2.1 Trial design

A randomized controlled trial will be conducted comparing the efficacy and noninferiority of a 12-week, web-based GSH CBT-E with the standard 20-week TAU CBT-E for BED at a specialized center for the treatment of eating disorders in the Netherlands (Novarum). Both treatments will be based on the CBT-E treatment protocol and are web-based. Assessments will take place at baseline (before randomization) and at four timepoints during and after treatment. Because the treatments vary in length, assessments will be synchronous in treatment phase rather than in the number of weeks since baseline. Web-based GSH CBT-E assessments will begin at the start of treatment (week 0), mid-treatment (week 5), end of treatment (week 12), 20 weeks post-treatment (week 32), and 40 weeks post-treatment (week 52). TAU CBT-E assessments will begin at the start of treatment (week 0), mid-treatment (week 5), end of treatment (week 20), 20 weeks post-treatment (week 40), and 40 weeks post-treatment (week 60; see **Table 1** for the breakdown of assessment timing). The duration of treatment and assessments in this study is 52 weeks for GSH and 60 weeks for TAU, making the total time horizon of the study 60 weeks (14 months). The study will be performed in line with the updated CONSORT guidelines for reporting parallel group randomized trials (39). To examine the cost-effectiveness and cost-utility of the web-based GSH CBT-E and TAU CBT-E, a complete economic evaluation will be conducted using ISPOR and CHEERS guidelines (40, 41). This study has been approved by the Medical Ethical Committee-United.

**Table 1:** Timing of planned assessments between groups.

	Weeks from baseline assessment	
Treatment group	GSH	TAU
T0: Baseline assessment	week 0	week 0
T1: Mid-treatment evaluation	week 5	week 5
T2: End of treatment	week 12	week 20
T3: 20 weeks post-treatment	week 32	week 40
T4: 40 weeks post-treatment	week 52	week 60

### 2.2 Participants

#### 2.2.1 Eligibility criteria

Participants must have a referral from their general practitioner, a DSM-5 diagnosis of BED or Other Specified Feeding and Eating Disorder – BED subtype (BED of subthreshold frequency and/or duration) as assessed by a psychiatrist or clinical psychologist, be ≥ 18 years, have a BMI between 19.5 kg/m^2^ ≤ 40 kg/m^2^, be sufficiently proficient in Dutch, have internet access and a computer or tablet, and provided informed consent to participate in the study. If a participant chooses to not participate in the study, they will be offered TAU CBT-E. Participants will be excluded if they have an eating disorder other than BED/Other Specified Feeding and Eating Disorder – BED subtype, acute psychosis, major depressive disorder, or suicidal ideation, as assessed via the Structural Clinical interview DSM-5 (SCID-5; 40, 42), have undergone any eating disorder treatment/intervention in the 6 months prior to their assessment, are pregnant, use recreational drugs (ex. marijuana, ecstasy) or take any medication that might influence eating behavior such as stimulant medications (ex. lisdexamphetamine), chlorpromazine, lithium, or olanzapine.

#### 2.2.2 Recruitment

Participants will be recruited from a Dutch eating disorder treatment center. Eligible patients will receive written and verbal study information and an informed consent form that will explain the research goals and provide information about their participation in the study. Patients will have two weeks to decide whether they want to participate in the study, during which time they can ask any questions they have regarding the study. Once an informed consent form has been signed and received, a phone appointment will be scheduled to conduct the baseline assessment. Participants will be informed that they can discontinue the study for any reason and at any time. To promote participant retention and follow-up, participants will be encouraged to contact the lead researcher or their therapist if they have any concerns, and they will receive a €20 gift card after completion of the final posttreatment assessment (week 40).

### 2.3 Interventions

#### 2.3.1 Therapists

All therapists are specialized in CBT-E treatment for eating disorders, have completed training by the Centre for Research on Eating Disorders at Oxford (CREDO), and have read *Overcoming Binge Eating* (36). To ensure a thorough understanding and adherence of administering web-based GSH CBT-E and TAU CBT-E, therapists will complete a two-day workshop and attend weekly 45-minute supervision sessions by the project leader (author BM) to ensure adherence to treatment plans and research protocols.

#### 2.3.2 Treatment as usual CBT-E

Treatment as usual CBT-E is the focused version of CBT-E, as it is indicated for patients with BED and exclusively addresses eating disorder pathology and is more effective than the broad version, which addresses external mechanisms (14). During the COVID-19 pandemic, face-to-face CBT-E treatment for BED could no longer continue, and consequently, video conferencing CBT-E was introduced as TAU at Novarum to ensure continued patient care. In this study, TAU CBT-E will be delivered through video conferencing over 20 sessions, each 50 minutes in length. The treatment follows the same structure and duration as face-to-face CBT-E; however, it is offered completely remotely. Video conferencing CBT-E has been as effective as face-to-face treatment (27, 31, 43, 44). Participants receiving TAU CBT-E will be required to monitor their eating behaviors, weigh themselves regularly, evaluate their progress, find alternative activities to binge eating, improve their problem-solving skills, plan for relapse prevention, and learn ways to cope through setbacks (36).

#### 2.3.3 Web-based guided self-help CBT-E

Web-based guided self-help CBT-E is an adapted and brief version of the focused form of CBT-E. The treatment is based on the guided self-help book *Overcoming Binge Eating* (36) is also available in Dutch *Overwin je eetbuien, waarom je te veel eet en hoe je daar mee kunt stoppen* (43), and is being built into a Dutch online application (Karify by Avinty). Before beginning web-based GSH, participants will be required to read part 1 of *Overcoming Binge Eating* (44). Treatment will be offered completely remotely using the web-based application, and focus on the same CBT-E principles as in TAU: monitoring eating behaviors, regular weighing, evaluating progress, finding alternative activities to binge eating, and improving problem-solving skills (36). Participants will receive weekly scripted feedback from a therapist during 12 phone sessions of 20 minutes. During these calls, therapists will review past assignments and discuss next steps in treatment.

### 2.4 Outcomes

#### 2.4.1 Primary outcome

The primary outcome will be the reduction in the number of binge-eating days in the previous 28 days between baseline and the end-of-treatment between groups. Binge-eating days will be defined as days on which one or more binge-eating episodes occurred, as reported by each participant. The primary outcome will be measured at the end of treatment.

#### 2.4.2 Secondary outcomes

Several secondary outcomes will be examined in this study, including full remission, body-shape dissatisfaction, therapeutic alliance, clinical impairment, health-related quality of life, attrition, and an economic evaluation using the costs associated with psychiatric illness, and treatment costs to assess cost-effectiveness and cost-utility, as described in detail below. The timing of secondary outcome measurements is outlined in **Table 2**. Baseline scores, demographic variables, and BMI will be examined as moderators.

**Table 2:** Timing of assessments and outcome measurements.

	Timepoints				
Outcome measure	T0: Baseline	T1: Mid-treatment	T2: End of treatment	T3: 20 weeks post-treatment	T4: 40 weeks post-treatment
Demographics	x				
EDE-Q	x	x	x	x	x
EDE	x	x	x	x	
TIC-P	x		x	x	x
EQ-5D-5L	x		x		
WAI		x	x		
CIA	x	x	x	x	x
BSQ	x	x	x	x	x

EDE-Q, Eating Disorder Examination- Questionnaire (EDE-Q); EDE, Eating Disorder Examination; TIC-P, Questionnaire on Costs associated with Psychiatric Illness; EQ-5D-5L, Health-Related Quality of Life questionnaire; WAI, Working Alliance Inventory; CIA, Clinical Impairment Assessment; BSQ, Body Shape Questionnaire.

##### 2.4.2.1 Full remission

Full remission will be assessed using the Eating Disorder Examination (EDE; 45) interview, with a combined measure of no binge-eating episodes in the previous 28 days, and eating disorder pathology below clinical cut-off of 2.8 (46). The EDE interview is a semi-structured interview assessing eating disorder pathology during the last 28 days, including binge eating. The scale consists of four subscales on dietary restraint, eating concern, weight concern and shape concern, and is measured on a 7-point Likert scale (47). The EDE has good internal consistency, discriminative validity, and reliability (48), and has been translated and validated in Dutch (49). The Eating Disorders Examination Questionnaire (EDE-Q; 50, 51) will be used at the 40-week post-treatment assessment. The EDE-Q is a 36-item questionnaire based on the EDE and the global score is calculated as the sum of the four subscales scores, divided by four (the number of subscales).

##### 2.4.2.2 Body shape dissatisfaction

Body shape dissatisfaction will be examined using the Body Shape Questionnaire (BSQ; 47). The BSQ contains 34 questions on a 6-point Likert scale ranging from “never” to “always”. The questionnaire has demonstrated good concurrent and discriminant validity, internal consistency, and test-retest reliability (52, 53).

##### 2.4.2.3 Therapeutic alliance

Therapeutic alliance, or the working relationship a patient has with a therapist, will be measured using Dutch version of the Working Alliance Inventory (WAI; 54, 55) to examine differences in therapeutic alliance between GSH and TAU, given therapeutic alliance can be impacted in online therapy (56). The WAI is a self-reported questionnaire with 36 items scored on a 7-point Likert scale examining the alliance between the patient and therapist. For the purposes of this study, only the patient’s perspective will be assessed. The questionnaire has three dimensions: Bonds, Tasks, and Goals. The Dutch version has been validated by (55).

##### 2.4.2.4 Clinical impairment

Clinical impairment refers to the psychosocial impairment suffered by patients as a direct result of their binge-eating disorder (Fairburn, 2008). The Clinical Impairment Assessment (CIA; 57) will be used to examine impairment secondary to eating disorder psychopathology, as the CIA differentiates between eating disorder pathology and secondary impairment due to the eating disorder. The questionnaire is self-reported and consists of 16 items with personal, social, and cognitive subscales, and is rated on a 4-point Likert scale.

##### 2.4.2.5 Health-related quality of life

Health-related quality of life will be measured using the Dutch version of the Euroqol EQ-5D-5L, a five-dimension, five level measure of health status (58). The scale has five dimensions with one item per dimension on mobility, self-care, usual activities, pain and discomfort, and anxiety/depression. Each item is rated on a 5-point scale with: no problems, slight problems, moderate problems, severe problems and extreme problems.

##### 2.4.2.6 Attrition

Attrition bias will be measured to determine whether participants in either group are more likely to have incomplete assessments or prematurely dropout of treatment. Attrition will be measured by the total amount of non-response and missing data (59), and dropout will be measured by the total number of participants that formally withdraw from treatment.

##### 2.4.2.7 Cost associated with a psychiatric illness

The costs associated with psychiatric illness will be measured using the Dutch version (60) of the questionnaire on healthcare consumption and productivity loss in patients with a psychiatric disorder (TiC-P) (61). The questionnaire for adults is composed of three parts and considers the cost of medical and mental health care, company doctors, social services, allied health care, residential care, alternative care, home help and support, addiction care, medication, and diet. The TiC-P will be used for the cost-effectiveness and cost-utility analysis to measure health care use and productivity loss.

##### 2.4.2.8 Cost of treatment

The cost of treatment will be determined from a healthcare perspective. For each patient, the cost of treatment will be calculated by multiplying standard Dutch cost prices (62) by the total number of minutes spent on direct patient contact. The time horizon for this study will be 52 and 60 weeks; therefore, no discounting for future costs will be applied.

##### 2.4.2.9 Cost-effectiveness and cost-utility

A cost-effectiveness analysis will be performed using the reduction in number of binge-eating days in the previous 28 days. A cost-utility analysis will examine the number of quality-adjusted life-years (QALYs) gained between randomization and post-treatment (63). Quality of life will be measured using the Dutch three-level variant of the EQ-5D (58). The Dutch tariff (64) will be used to translate EQ-5D-5L scores into health utilities, and utility weights will be assigned which reflect the patient’s health between death (0) and perfect health (1) (65). One QALY reflects one year in perfect health. The incremental cost-effectiveness ratio (ICER) will be calculated using the costs and effects of treatment. Confidence intervals of the ICER will be calculated using non-parametric bootstraps to provide incremental costs, effects, and a cost-effectiveness ratio. Data on resource use (health care uptake) and productivity losses will be collected with a Dutch version of the TiC-P (60, 61).

#### 2.4.3 Moderators

Baseline scores, demographic variables, and BMI will be examined as moderators. Baseline scores, including the number of objective binges and eating disorder severity will be examined given prior research suggests that more severe BED pathology at baseline predicts less improvement in BED pathology at the end of CBT-E treatment (66, 67), and may also predict greater treatment dropout (21). Demographics, such as age, gender, and level of education, will be examined as possible moderators. Lastly, BMI, calculated by dividing the participant’s weight in kilograms by the square of height in meters (kg/m2), will be examined as a moderator.

### 2.5 Statistics

#### 2.5.1 Sample size and power estimation

The primary objective of the proposed study is to determine whether GSH is noninferior to TAU in efficacy. It is expected that both groups will have a reduction in the number of binge-eating days, and GSH is expected to be noninferior compared to TAU in the reduction of binge-eating days in the previous 28 days at the end of treatment.

To calculate the sample size for this noninferiority trial, the significance level was set at *α* = 0.05, power was aimed at 80%, and the noninferiority margin (*Δ*) was set to 1 (*SD* = 2.4) binge-eating day (68) in the previous 28 days between groups. With these assumptions, *n* = 144 (*n* = 72 per arm) participants will be needed to determine noninferiority. The noninferiority margin and standard deviation are based on noninferiority margins from prior studies examining online treatments to reduce eating disorder behaviors (68, 69) and clinical experts on BED treatment outcomes. Efficacy studies of CBT-E (70) and internet-based guided self-help for BED (68) report a 20% dropout; therefore, 20% more participants will be included to compensate for dropout. The sample size corrected for dropout is *N* = 180 (*n* = 90 per arm); therefore 180 participants will be randomized. This sample size calculation was performed using the R package ‘SampleSize4ClinicalTrials’ (71–73) based on a continuous outcome, noninferiority design.

#### 2.5.2 Randomization

Randomization will be performed using a 4, 6, 8 block design by Castor Electronic Data Capture (Amsterdam, the Netherlands, 2019) and will take place after the baseline assessment. The allocation ratio will be 1:1. This software will ensure the concealment of allocation and blinding. Stratification will take place based on a BMI of 19.5 ≤ 35 or 35.1 ≤ 40.

#### 2.5.3 Statistical analyses

To measure the efficacy of treatment on the reduction of binge-eating days, the number of binge-eating days will be reported as means (standard deviation) between groups and analyzed using a general linear mixed model. Effect sizes will be calculated using Cohen’s *d* (0.2 small, 0.5 medium, 0.8 large; 74) and adjusted for bias (75). Sensitivity analyses will be conducted to test the robustness of the results; given the 20% anticipated dropout rate, analyses will be run on an intention-to-treat protocol, and multiple imputations will be used to handle missing data. To examine possible treatment moderators, a regression analysis with subgroups will be performed by analyzing interaction terms between the moderators and treatment group. Data will be nested within BMI groups (19.5 ≤ 35 and 35.1 ≤ 40) and include random and fixed effects. If significant interactions exist, *post-hoc* analyses will be performed to further explore the moderation.

The cost-effectiveness and cost-utility analyses will be performed from a healthcare perspective. The cost-effectiveness analysis will examine the reduction in binge-eating days as the measure of effect measure, and the cost-utility analysis will use QALYs as effect measure. Differences in costs and effects between groups will be calculated as the difference in cumulative direct costs using the reduction of binge-eating days as the outcome measure. ICERs will be calculated as ICER = (Costs GSH − Costs TAU)/(Effects GSH – Effects TAU) with reduction in binge-eating days as the effect. ICERs to report comparative differences in QALYs will be calculated as ICER = (Costs GSH − Costs TAU)/(Effects GSH – Effects TAU). The costs, effects, and ICERs will be plotted on a cost-effectiveness plane to demonstrate the differences between the costs and effects of both groups, with TAU positioned at the origin of the cost-effectiveness plane. Cost-effectiveness acceptability curves will be used to examine the probability that the cost-effectiveness of GSH is equally as effective with lower costs compared to TAU by a willingness-to-pay for each unit of effect (reduction in binge-eating days or QALYs). The willingness-to-pay for each additional unit of effect in the Netherlands ranges between €20,000 and €80,000 per QALY (25, 76) and €22–€110 per binge-free day in the United Kingdom and the United States (31). All statistical analyses will be conducted using R (77) and SPSS (78).

#### 2.5.4 Data monitoring

All data will be stored in the secure data storage environment within the primary institute and will only be accessible to relevant senior researchers and the project leader with secure login procedures. All data will be handled confidentially, and each participant will be provided with an identification code to separate their personal information from their data, in line with the EU General Data Protection Regulation (79) and the Dutch Act on Implementation of the General Data Protection Regulation (Uitvoeringswet *AVG, UAVG).* Data will be monitored by a statistician and data steward independent from the study, who will review the data collected to ensure the validity of the study findings. Any significant harmful events or reactions possibly relating to the intervention will lead to unblinding. The study will be temporarily halted if there is sufficient ground to suggest that continuing the study could jeopardize the health or safety of a participant. Any participant exhibiting a psychiatric crisis will be assessed and treated accordingly, and any serious adverse events will be reported to the METC. Finally, posttrial support will be offered to participants who require it.

## 3 Anticipated results

It is expected that at the end of treatment, the difference in reduction of binge-eating days between web-based GSH CBT-E and TAU CBT-E is within the noninferiority margin (Δ) of 1 binge-eating day (68). Regarding secondary outcomes, it is expected that there will be no difference in the rate of full remission, clinical impairment, or reduction in body shape dissatisfaction between groups. However, it is expected that more severe binge-eating pathology at the start of treatment will predict a weaker treatment response, regardless of condition (80). In addition, the economic evaluation is expected to demonstrate that GSH is more cost-effective than TAU, while having similar cost-utility (31, 81). Therapeutic alliance is expected to be the similar between groups (82). Attrition rate is expected to be approximately 20% in both conditions (68, 83). As for moderators, it is expected that baseline scores will moderate the treatment effect; for example, it is expected that participants with a greater number of objective binges at baseline will demonstrate a slower response to treatment (84). Demographic information is expected to have no effect on the reduction of binge-eating days. Lastly, is expected that BMI will have a small moderating effect on binge-eating reduction, such that participants with a greater baseline BMI will have a weaker response to treatment, regardless of group.

## 4 Discussion

This protocol outlines a randomized controlled trial comparing clinical and economic outcomes between two web-based treatments for BED: web-based GSH CBT-E and TAU CBT-E. This study will be the first to directly compare the efficacy of a low-intensity and low-cost alternative compared to TAU CBT-E alongside a complete economic evaluation. Furthermore, this study will establish whether the improvements seen in 20 weeks of TAU CBT-E can be achieved in 12 weeks of web-based GSH CBT-E. The aim of the proposed study is to determine whether web-based GSH CBT-E is noninferior to TAU CBT-E in reducing the number of binge-eating days at the end of treatment. Additional outcomes including full remission, body shape dissatisfaction, therapeutic alliance, clinical impairment, health-related quality of life, attrition, and an economic evaluation to assess the cost-effectiveness and cost-utility of web-based GSH CBT-E and TAU CBT-E will be evaluated to provide a thorough understanding of the efficacy and effectiveness of web-based GSH CBT-E and for whom it is best indicated. Baseline scores, demographic variables, and BMI will be examined as moderators to support the development of a personalized approach to treatment and to optimize the allocation of treatment resources.

The findings from this study will offer compelling insight into whether BED can be treated using a stepped-care model, whereby web-based GSH CBT-E is offered as the recommended first line of treatment for patients in the Netherlands. In this model, if a patient does not improve after web-based GSH CBT-E, they will be offered the higher intensity video conferencing CBT-E. If web-based GSH CBT-E is noninferior to TAU CBT-E in reducing the number of binge-eating days, the treatment can be widely disseminated among allied healthcare workers who identify a patient with BED. This will allow more patients to receive timely support and shorten treatment waitlists, ultimately supporting more patients in their BED recovery.

## Ethics statement

The study has been approved by the Medical Research Ethics Committees United on May 25th, 2021, case number NL76368.100.21. The participants will provide their written informed consent to participate.

## Author contributions

EvB: Writing – original draft. BM: Writing – review & editing. MdJ: Writing – review & editing. JP: Writing – review & editing. EvdB: Writing – review & editing. EdB: Writing – review & editing.
